# Path Model Factors Associated with Depressive Symptoms among Older Thais Living in Rural Areas

**DOI:** 10.3390/geriatrics7030069

**Published:** 2022-06-16

**Authors:** Inthira Roopsawang, Suparb Aree-Ue, Surinrat Baurangthienthong, Jansudaphan Boontham, Yuwadee Phiboonleetrakun

**Affiliations:** 1 Ramathibodi School of Nursing, Faculty of Medicine Ramathibodi Hospital, Mahidol University, Bangkok 10400, Thailand; inthira.ros@mahidol.edu; 2 Kuakarun Faculty of Nursing, Navamindradhiraj University, Bangkok 10400, Thailand; surinrat.bau@nmu.ac.th; 3 Private Clinic, Chiang Mai 50000, Thailand; jansudaphan.b@nmu.ac.th; 4 Private Clinic, Bangkok 10400, Thailand; yuwadee.phi@gmail.com

**Keywords:** community-dwelling older adults, depressive symptoms, older Thais, frailty, locomotive syndromes

## Abstract

Depressive symptoms are complex and are often more severe in older people. However, there is limited research exploring the causal relationships between depression and its associated factors in the geriatric population, particularly in Thailand. We aimed to evaluate the direction of these complex relationships in the Thai population. A cross-sectional design was conducted on 312 Thai community-dwelling older adults aged 60 years or above who registered for primary care services. The participants were recruited from July 2019 to January 2020, and they responded to standard assessments. The relationships between pain, the number of medications, frailty, locomotive syndrome, and depressive symptoms were investigated using path analysis. The results showed that most participants were women and had multiple diseases, mild pain, frailty, and grade I–II locomotive syndrome. The prevalence of depressive symptoms was 16%. The model showed significant positive direct and indirect paths from locomotive syndrome to depressive symptoms (β = 0.296, *p* < 0.01; β = 0.099, *p* < 0.01, respectively). There was a significant positive direct path from frailty to depressive symptoms (β = 0.219, *p* < 0.01) and a significant positive indirect path from pain to depressive symptoms (β = 0.096, *p* < 0.01).

## 1. Introduction

Moderate depressive symptoms are common and are frequently invisible, resulting in their difficult management in aging populations [1]. The estimated prevalence of depressive symptoms in the global population of older adults (65 years or older) is 10–15%; however, most individuals with such symptoms are unlikely to receive appropriate management [2]. According to recent predictions, approximately 8 out of 10 older people worldwide will live in developing regions by 2050 [1,2]. The effective management of depression varies depending on the healthcare systems and geographical location [1], resulting in increased inequity in mental healthcare. Age-related decline is the most common preexisting problem in individuals who develop depressive symptoms and dementia [3]. The sequelae of depressive symptoms continue to increase, leading to poor health outcomes, barriers to seeking healthcare, and greater future medical care costs [4,5]. Depressive symptoms are more severe in older people, for whom better prevention is required. Therefore, the identification of factors that prevent or affect depressive symptoms is a key strategy to enhance equity, provide better care, and contribute to global strategic plans for promoting healthy aging [6].

According to studies of community-dwelling older adults, unidentified or untreated depressive symptoms have substantial effects on psychological, behavioral, and physical aspects throughout life [7,8]. In attempts to enhance care, several factors related to depressive symptoms and their progression have been investigated, including pain, chronic disease, sedentary behavior, disability, cognitive impairment, and socioeconomic status [9,10,11]. The geriatric syndrome of frailty is an important predictor of psychological health, particularly depressive symptoms [9,12,13]. The functional decline caused by the aging process is also strongly associated with depressive symptoms [3]. Recently, a few studies have shown that motor function declines with age—locomotive syndrome (LS)—and this is associated with depressive symptoms in community-dwelling older adults [14]. Although many studies have identified the factors associated with depressive symptoms, more research is needed to explore the direction and causality of such associations, particularly across healthcare contexts. There are substantial healthcare barriers and gaps in care and research regarding the relatively new areas of frailty and LS [15,16,17]; very little attention has been paid to the roles of geriatric factors and depressive symptoms in low–middle-income countries [18].

Thailand is a low–middle-income country and is one of the five Asian countries with the highest population aging rate. Therefore, it is likely that management of the consequences of depressive symptoms in older adults will become a problem in Thailand. Some research has been carried out on the causal relationships between depressive symptoms and other factors in community-dwelling older adults; however, the direction of these relationships and the effect of geriatric factors on depression remain speculative in the Thai context. In addition, the overlapping mechanisms that underpin the effect of frailty on depression have not been fully explored [19]. A more in-depth understanding of the factors associated with depressive symptoms is warranted to provide better care and promote equity.

As the population ages, inquiring about the factors influencing depressive symptoms has become more interesting; however, few empirical investigations have explored the impact of geriatric factors—frailty and LS—on this population. Thus, the present study aimed to explore the relationships between pain, the number of medications used, LS, frailty, and depressive symptoms in Thai community-dwelling older adults. To our knowledge, this study is the first to explore the direction of the associations of frailty and LS with depressive symptoms. The findings may provide a foundation for strategies to ameliorate depressive symptoms, develop care interventions, and enhance the quality of life in this population.

## 2. Methods

### 2.1. Study Design

This was a cross-sectional study that used path analysis. The study was conducted with community-dwelling older adults in Thailand from July 2019 to January 2020. A simple random method with purposive criteria was used to recruit participants from the six regions of Thailand.

### 2.2. Participants and Recruitment

A simple random method was used to select three of the six regions of Thailand. One province was randomly chosen to represent a selected region; then, five subdistrict primary care settings were randomly chosen for each selected province. Eligible participants were recruited from the registries of the subdistrict primary care settings. Adults aged 60 years or older who visited those settings were randomly enrolled (one person: 10 persons) without replacement. The inclusion criteria were (1) aged 60 years or over, (2) the ability to understand and communicate in Thai, and (3) consent for participation in the study. The exclusion criteria were (1) bedridden, (2) cognitive impairment (Mini-Cog scores < 3) [20], and (3) diagnosed with or presenting severe depression or psychiatric disorders; these older adults were identified, given advice, and referred to proper care in their primary care setting. Based on the recommendations, the sample is therefore recommended to be 20 samples per estimated parameter; thus, the initial sample of 260 persons was required. Moreover, 20% of the initial sample was added to increase valid prediction; the final total of participants was 312 older persons.

### 2.3. Ethics Statement and Procedures

The study was conducted after obtaining institutional review board ethical approval (ID: MURA2019/143) and written consent and/or oral permission from the participants or their family members/caregivers. The participants were interviewed by trained research assistants (five experienced registered nurses) for 30–40 min about their general health status and outcomes of interest. The participants who were not comfortable responding to or completing the questions could leave the study at any time with no consequences as to their treatment or access to healthcare services. All participant information was kept confidential in accordance with the participants’ personal rights, dignity, and privacy.

### 2.4. Measures

The Mini-Cog Thai version [20] was used to assess cognitive status. This Thai version of the scale was translated from the original Mini-Cog, which is used to screen for cognitive impairment and dementia. The Mini-Cog test has two parts: (a) three-item recall; (b) clock drawing. The total possible score is 5, and a total score of less than 3 indicates a greater likelihood of cognitive impairment or dementia. Higher scores indicate a lower likelihood of cognitive impairment or dementia. The Thai version of the Mini-Cog has shown good interrater reliability (K = 0.80, 95% confidence interval 0.50–1.00, *p* < 0.001) and positive concurrent validity with the Mini-Mental Status Exam Thai 2002 (r = 0.47, 95% confidence interval 0.37–0.55, *p* = 0.007) [20].

The 25-question Geriatric Locomotive Function Scale (GLFS-25) Thai version [21] was used to assess LS. This scale has been translated into Thai from the original GLFS-25 [22], which consists of 25 questions: (1) pain during the previous month (four questions), (2) pain during the activities of daily living during the previous month (sixteen questions), (3) social function (three questions), and (4) the participant’s mental status during the previous month (two questions). Each item is rated on a 5-point scale ranging from no impairment (0) to severe impairment (4); the total possible score ranges from 0 to 100. The total score is used to categorize respondents into three LS grades: No LS (scores of 0–6; normal), Grade I LS (scores of 7–15), and Grade II LS (scores of ≥16). The GLFS-25 has demonstrated excellent reliability (test–retest interclass correlation = 0.71–0.92) [22] and good predictability for LS in older adults with orthopedic conditions (sensitivity = 0.75–0.84; specificity = 0.89–0.92) [23]. The GLFS-25 Thai version has been tested and has demonstrated good validity (content validity index = 1) and reliability (Cronbach’s alpha = 0.94 – 0.95) [21].

The Numeric Rating Scale (NRS), a standard self-rating pain scale, was used to assess pain intensity. The total scores on this scale range from 0 to 10 and are categorized as no pain (0), mild pain (1–3), moderate pain (4–6), and severe pain (7–10). This scale has demonstrated good reliability and validity for measuring pain intensity [24].

The Reported Edmonton Frail Scale Thai version (REFS-Thai) is a self-report instrument that was translated from the Reported Edmonton Frail Scale (REFS). The scale defines frailty using an accumulation deficit model. This measure has been validated for use in Thai inpatient settings [15]. The REFS-Thai evaluates nine domains: general health status, cognitive function, functional independence, continence, medication use, nutrition, mood, social support, and self-performance. The total possible score on the REFS-Thai version is 18. The scores are categorized as non-frailty (0–5), apparent frailty (6–7), mild frailty (8–9), moderate frailty (10–11), and severe frailty (12–18). The REFS-Thai has shown acceptable internal consistency (Cronbach’s alpha = 0.73) and good interrater reliability (linear weights K = 0.87, *p* < 0.001) when administered by personnel with non-geriatric training.

The 15-item Geriatric Depression Scale Thai version (TGDS-15), a short-form screening tool [25], was used to assess major depressive episodes in older adults. This tool was translated into Thai from the original scale. The TGDS-15 consists of 15 questions with binary responses (yes/no), which are scored as 0 and 1. The maximum total score is 15. The cut point for depression is a score of ≥5. The TGDS-15 has been tested in Thai older adults and has demonstrated acceptable reliability (Cronbach’s alpha = 0.85) and excellent predictive value in detecting depression (sensitivity = 0.86, specificity = 0.91) [25]. In the present study, participants who presented with depressive symptoms (TGDS-15 score ≥ 5) received advice on basic symptom management, access to care, and useful resources. Such participants and/or their families were asked for their permission for referral or specific monitoring for risk prevention and continuing care.

### 2.5. Data Analysis

STATA version 15.1 (Licensed to Faculty of Medicine Ramathibodi Hosipital) was used to analyze participants’ demographic data and to perform pathway analyses to examine the associations of multiple diseases, pain, frailty, and LS with depressive symptoms. A *p*-value < 0.05 was considered significant. The model fit indices were tested using the χ^2^ goodness-of-fit test, the comparative fit index (CFI), the Tucker–Lewis index (TLI), the root mean squared error of approximation (RMSEA), and the standardized root mean squared residual (SRMR). Values of CFI ≥ 0.95, TLI ≥ 0.95, RMSEA < 0.08, and SRMR < 0.05 demonstrated an acceptable fit.

## 3. Results

### 3.1. Participant Demographics

A total of 312 community-living older adults participated; of these, 217 were women (69.6%). The mean age of the participants was 69.66 years (standard deviation = 6.95; range = 60–90 years). Approximately half of participants (56.4%) were married. Most participants (81.1%) had received primary school education. Approximately one-third of the participants (33.7%) reported sufficient incomes, which were mostly provided by their children (42.6%). Most participants (78.2%) had chronic illness; hypertension was the most prevalent illness (59.9%), followed by dyslipidemia and diabetes (43.9% and 26.3%, respectively). Nearly half of the participants were overweight (48.4%), followed by normal weight. Few participants reported a family history of depression (1.6%); 87.2% of participants had depressive symptoms. Frailty and non-frailty showed a similar prevalence (49.4% and 50.6%, respectively). The prevalence of LS grade I and grade II was 35.3% and 32.4%, respectively. Table 1 shows detailed participant characteristics.

### 3.2. The Association and Direction of Depressive Symptoms

Table 2 illustrates the mean scores for the study variables: the number of medications, pain intensity, frailty, LS, and depressive symptoms. Table 3 is a correlation matrix showing the correlations among these variables.

**Figure 1:**
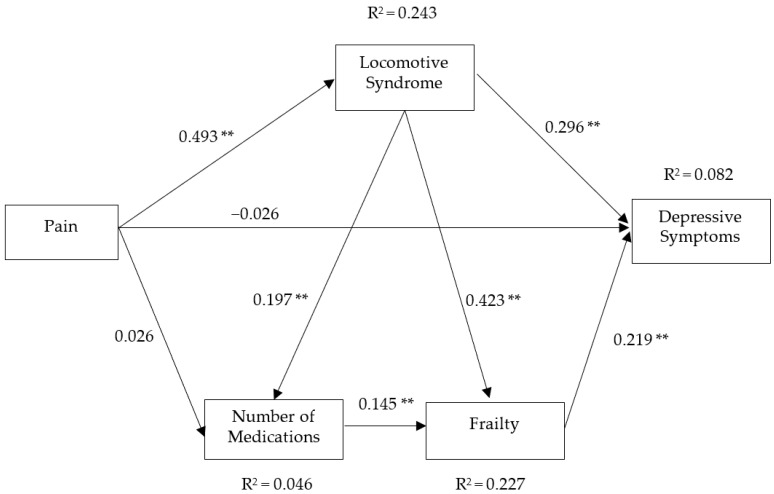
Path model of factors associated with depressive symptoms among older people. Overall model fit, χ2 = 1.759, *p* = 0.6239, comparative fit index = 1.000, Tucker–Lewis index = 1.000, root mean squared error of approximation = 0.000, standardized root mean squared residual = 0.017. Significant at ** *p* < 0.01.

Figure 1 shows the results of the path analysis. The model showed significant positive direct and indirect paths from LS to depressive symptoms (β = 0.296, *p* < 0.01; β = 0.099, *p* < 0.01, respectively). There was a significant positive indirect path from pain to depressive symptoms and a significant positive direct path from frailty to depressive symptoms. Additional information about the path analysis results is shown in Table 4.

## 4. Discussion

To our knowledge, this is the first study to investigate the associations of LS, the number of medications, and frailty with depressive symptoms in older Thai adults and examine the magnitude and direction of these associations. Pain and frailty showed mild significant associations with depressive symptoms, whereas LS demonstrated a moderate association. Notably, pain, frailty, and LS were moderately associated. Regarding the direction of the complex relationships among these factors, our findings clarify the links between age-related syndromes, physical status, and depressive symptoms; frailty was a significant direct predictor of depressive symptoms, whereas pain was a significant indirect predictor. Only LS demonstrated positive relationships—both direct and indirect— with depressive symptoms. The number of medications had a significant direct effect on frailty but demonstrated both direct and indirect effects on pain. Consistent with the literature, our findings confirm the complex effects of chronic illness, pain, and age-related decline on depressive symptoms [9,10,11]; therefore, it can be assumed that older persons experiencing multiple comorbidities, pain, frailty, and LS are more likely to develop depressive symptoms.

The path analysis findings showed that pain, frailty, and LS directly predicted depressive symptoms. These findings are in line with previous evidence indicating that physical status and age-related decline affect mental health, particularly depressive symptoms [3,7,8]. Pain demonstrated a significant positive indirect effect (β = 0.096, *p* < 0.01), but a negative direct effect (β = −0.026), on depressive symptoms. Contrary to expectations, we did not find a significant direct effect of pain on depressive symptoms, but the observed negative direct effect of pain on depressive symptoms is noteworthy. This finding indicates that participants with lower pain intensity are more likely to have greater depressive symptoms; these findings are very encouraging because they suggest that pain may not independently predict depressive symptoms in older adults with multiple comorbidities. A recent study by Gunnarson and colleagues showed that the identification of specific pain characteristics (pain-related daily activities and pain location) is a better predictor of both anxiety and depressive symptoms than pain intensity [26].

The finding of significant indirect effects of pain, the number of medications, and frailty on depressive symptoms is consistent with the findings from many previous studies, including a recent study [27] demonstrating that pain and frailty may form a synergic factor that affects both physical limitations and depression. Age-related musculoskeletal conditions vary in severity according to the levels of inflammation; this degenerative process causes pain that hinders physical function and movement and results in frailty [28]. A synergy between pain and frailty in older adults has been reported in the literature; as skeletal muscle use declines owing to persistent pain, muscle mass loss and functional impairment increase, which considerably accelerates frailty [28,29,30]. Additionally, there is an association between frailty and greater chronic pain and functional limitation in older adults [31]. Physical and mental health are also strongly related [3,9,10,11]; chronic pain and frailty are associated with mental health problems such as depressive symptoms. Personal factors such as multicomorbidities and obesity may also induce pain and frailty [32], which ultimately increase the risk of depressive symptoms [9,13,33]. The previously observed association between pain and multiple comorbidities accords with the findings of the present study, which demonstrated both direct (β = 0.026) and indirect effects (β = 0.099) of the number of medications on pain. Interventions for frailty in older adults with chronic pain are needed to prevent depressive symptoms [34]. However, associations between pain, frailty, and depressive symptoms seem common in older people. Some studies suggest that more investigation of the mediating effect of pain and frailty on depressive symptoms is needed [33,35]. Notably, our finding of an indirect effect of frailty on pain and depressive symptoms suggests that the interrelationships between pain and depressive symptoms may be mediated by other factors; thus, future work is needed to investigate the mediating effect of frailty on depressive symptoms.

The present findings increase our knowledge of frailty and depressive symptoms. As in previous studies [9,12,13], we found a significant positive direct effect of frailty on depressive symptoms. Most of our participants were not in the moderate–severe frailty range (average REFS score = 2.43). Therefore, the present findings provide strong evidence that prefrailty (apparent frailty) or mild frailty predicts depressive symptoms in older adults. Although we found a similar prevalence of frailty and non-frailty, many factors (e.g., poor physical function, uncontrolled pain, multiple comorbidities, poor nutrition) may accelerate the frailty processes [7,8,9,10]. In accordance with the present results, a recent study demonstrated that the risk factors that affect frailty processes in the prefrail population are gender, the activities of daily living limitations, the number of chronic diseases, the number of medications, and obesity, particularly in older adults living alone [36]. As the development of frailty is a dynamic process, prefrailty is likely to develop into frailty. There is evidence that frailty is a coexisting condition that substantially affects both physical and mental health in older adults [9,13]. The prevalence of frailty has increased; however, there is a lack of appropriate measurement instruments and a lack of an understanding of frailty in some countries, resulting in the difficulty of identifying the condition [15,16]. Thus, our findings may help to increase knowledge of frailty and frailty assessment to enhance care equity in this population. Importantly, the early identification of prefrailty and the management of the factors that affect frailty processes may be necessary to prevent depressive symptoms in older adults. Further studies that take these variables into account are needed.

We found that LS had a significant positive direct and indirect effect on depressive symptoms; this findings are consistent with previous studies on community-dwelling older adult populations [14]. LS showed a strong relationship with depressive symptoms, indicating that older adults with LS were more likely to develop depressive symptoms. Greater locomotive functional impairment is associated with worse depressive symptoms. Previous studies have shown that LS strongly predicts depressive symptoms in older adults [14]; moreover, pain, physical limitations, and obesity strongly affect the severity of LS [3,37]. The present findings of a direct effect of LS on pain support previous findings. An important issue emerging from these findings is that LS directly affected the number of medications but had both direct and indirect effects on frailty. It is possible that these results reflect age-related conditions that are antecedents of LS [17,37], because frailty and the number of medications indicate both the aging process and the presence of chronic disease. Evidently, the trajectory of chronic disease is likely to result in frailty; moreover, frailty is a geriatric syndrome that causes functional impairment throughout the body [16]. Although the relationship between frailty and LS is unclear, frailty also affects locomotive function and organs, bones, joints, muscles, and ligaments. Some studies suggest that frailty is a concomitant of LS [14,17] because physical impairment and reduced mobility are clinical presentations of both frailty and LS. Our findings support these previous studies because we found that most participants had LS stages I and II. Therefore, the present findings not only demonstrate complex associations among the variables examined but also may increase the understanding of the direction of the relationships between geriatric syndrome factors (frailty and LS) and depressive symptoms in older adults.

Overall, our model demonstrated good fit indices and showed that the main factors that affect depressive symptoms were pain, the number of medications, frailty, and LS, which explained a large percentage (76%) of the variability in depressive symptoms. Most participants experienced mild pain, multiple diseases, frailty, and LS grades I–II; approximately 16% had depressive symptoms but no family history of depression. These results suggest that age-related decline and physical impairment have direct effects on depressive symptoms in older adults.

Although plausible associations were demonstrated, the study had some limitations. First, most participants were women, so the findings may not be generalizable to the whole population. Second, analyses of other causal and mediating effects are warranted. Finally, because this was a cross-sectional study, the magnitude of the effects of the predictors must be interpreted with caution. Despite these limitations, the study findings suggest that the prevention of depressive symptoms requires multiple strategic approaches, informed by the knowledge of gerontological care, to manage geriatric factors.

## 5. Conclusions

The present study provided novel insights into the effect of geriatric factors on depressive symptoms in a low–middle-income country. Depressive symptoms in older adults are caused by complex interactions between multiple diseases in terms of the number of medications used, pain, frailty, and LS. Comprehensive interventions that integrate gerontological knowledge—specifically regarding the complex interactions among these study variables—should be provided for individuals with these conditions. In addition, it is essential to increase the screening for frailty and LS since these two factors are new geriatric syndromes influencing depressive symptoms. Further studies on the role of frailty and LS in the long-term monitoring of older adults would be useful.

## Figures and Tables

**Table 1:** Characteristics of study participants (*n* = 312).

Variables	*n* (%)	Variables	*n* (%)
Gender		Family History of Depression	
Women	217 (69.6)	No	307 (98.4)
Men	95 (30.4)	Yes	5 (1.6)
Marital Status		Chronic Illness	
Married	176 (56.4)	Yes	244 (78.2)
Widowed/divorced	120 (38.5)	No	68 (21.8)
Single	16 (5.1)	Type of comorbidity *	
Education		Hypertension	187 (59.9)
Illiterate	12 (3.8)	Dyslipidemia	137 (43.9)
Primary school	253 (81.1)	Diabetes	82 (26.3)
Secondary school	25 (8.0)	Coronary disease	31 (9.9)
Bachelor’s degree or higher	7 (2.2)	Other (e.g., chronic kidney disease, carcinoma)	11 (3.5)
Income		Medication Use	
Sufficient	105 (33.7)	Yes	233 (74.7)
Insufficient	207 (66.3)	No	79 (25.3)
Source of Income		Type of Medication Taken *	
Children	133 (42.6)	Antihypertensive drugs	183 (58.7)
Work	82 (26.3)	Hypoglycemic drugs	75 (24.0)
Savings	36 (11.5)	Vasodilators	21 (6.7)
Other	61 (19.6)	Antidepressants	19 (6.1)
Living Conditions		Hypolipidemic agents	145 (46.5)
With children	126 (40.4)	Analgesics/painkillers	14 (4.5)
With spouse	155 (49.7)	Antacids	23 (7.4)
Alone	29 (9.3)	Antiplatelet drugs	57 (18.3)
With cousin	2 (0.6)	Other (e.g., antithyroid, gout)	52 (16.7)
Body Mass Index (kg/m^2^) ^a^		Frailty	
Underweight (<18.5)	26 (8.3)	Non-frail	158 (50.6)
Normal (18.5–22.9)	116 (37.2)	Apparent–mild	140 (44.9)
Overweight (23–29.9)	151 (48.4)	Moderate–severe	12 (3.8)
Obesity (≥30)	19 (6.1)	Locomotive Syndrome	
Depressive Symptoms		Normal (0–6)	101 (32.4)
No (0–5)	46 (14.7)	Grade I (7–16)	110 (35.3)
Yes (≥5)	266 (85.3)	Grade II (≥16)	101 (32.4)

* More than one answer; ^a^ Asian index value.

**Table 2:** Descriptive analysis of the study variables (*n* = 312).

Variable	Possible Scores	Actual Scores	M ± SD
Number of Medications	0–∞	0–14	2.95 ± 2.74
Pain Intensity	0–10	0–10	2.93 ± 2.55
Frailty	0–18	0–13	5.46 ± 2.43
Locomotive Syndrome	0–100	0–86	14.38 ± 14.12
Depressive Symptoms	0–15	0–15	11.20 ± 4.48

M = mean; SD = standard deviation.

**Table 3:** Correlation matrix for study variables (*n* = 312).

Variables	1	2	3	4	5
1. Number of Medications	1				
2. Pain	0.12 *	1			
3. Frailty	0.23 **	0.20 **	1		
4. Locomotive Syndrome	0.20 **	0.49 **	0.45 **	1	
5. Depressive Symptoms	−0.06	−0.13 *	0.08	−0.21 **	1

Significant at * *p* < 0.05 and ** *p* < 0.01.

**Table 4:** Standardized direct and indirect effects of exogenous and endogenous variables.

Variables	Number of Medications		Frailty		Locomotive Syndrome		Depressive Symptoms	
	DE	IE	DE	IE	DE	IE	DE	IE
Pain	0.026	0.099 **	-	0.227 **	0.493 **	-	−0.026	0.096 **
Locomotive Syndrome	0.197 **	-	0.423 **	0.029 *	-	-	0.296 **	0.099 **
Number of Medications	-	-	0.145 **	-	-	-	-	-
Frailty	-	-	-	-	-	-	0.219 **	-

Significant at * *p* < 0.05 and ** *p* < 0.01. DE = direct effect; IE = indirect effect.

## Data Availability

Not applicable.
